# Usage and Perceptions of Electronic Patient Records Experienced by Users and Nonusers in the Canton of Vaud, Switzerland: Mixed Methods Study

**DOI:** 10.2196/83702

**Published:** 2026-04-21

**Authors:** François Bastardot, Catherine LaRocco, Mathilde Robert, Veronika Schoeb

**Affiliations:** 1 Department of Medical Informatics, Lausanne University Hospital (CHUV), Lausanne, Switzerland; 2 Department of Research & Innovation, HESAV School of Health Sciences - Vaud, HES-SO University of Applied Sciences and Arts Western Switzerland, Avenue Beaumont 21, Lausanne, 1011, Switzerland, 41 215566256

**Keywords:** electronic patient record, mixed methods study, health literacy, digital health literacy, health disparities, patient engagement, Swiss health care system, patient-provider relationship, communication, perceptions

## Abstract

**Background:**

Electronic patient records (EPRs) have shown potential to improve health care delivery, coordination, and patient engagement. In Switzerland, the development of a national EPR is supported by the Confederation, while its deployment is carried out by the cantons through EPR communities. Since health policy and the organization of health systems are entrusted to cantonal governments, each of the 26 Swiss cantons must decide its strategy for the implementation and application of EPRs within its jurisdiction. The canton of Vaud has joined with other French-speaking cantons (Geneva, Valais, Fribourg, and Jura) in organizing the implementation of the EPR through the CARA association since 2018. By June 2025, approximately 32,350 individuals have opened an EPR (Dossier électronique du patient) through CARA, accounting for 28% of all EPRs opened nationwide at that time.

**Objective:**

This study aimed (1) to document patients’ use of EPR in the canton of Vaud and to examine the user profiles; and (2) to explore users’ and nonusers’ perceptions regarding EPR benefits and barriers.

**Methods:**

This mixed methods study was conducted in 2 phases. First, a quantitative questionnaire examined EPR user profiles (eg, demographics and health status). Health literacy and digital health literacy were assessed using the HLS19-Q12-CH (Health Literacy Survey 2019-2021) and HLS19-DIGI-CH (Digital Health Literacy) scales in French. Second, qualitative semistructured interviews explored experiences and perceptions of users and nonusers through thematic analysis. Data from both phases were integrated during interpretation. All analysis was conducted in French with quotes translated for publication.

**Results:**

The population included 839 patients with EPRs (early adopters from December 2021 to December 2023) and a matched control group. Overall participation was 19.3% (324/1678), with higher participation among EPR users (255/839, 30.4%) than nonusers (69/839, 8.2%). Early adopters were predominantly male (185/255, 72.5%), highly educated (155/255, 60.8%), affected by chronic illness (204/255, 80%), and with extensive health care networks (80% consulted 2 or more health care professionals in the last 3 months). The vast majority (249/253, 98.4%) had a general practitioner. While only a minority (76/252, 30.2%) of early adopters were satisfied with the service provided by the EPR, a majority (172/254, 67.7%) would recommend it to their family and friends. Qualitative analysis identified themes that influence EPR adoption, including the contextual environment, level of health literacy, EPR as a tool, professionals’ resistance to EPR, and level of engagement with digitalization.

**Conclusions:**

This study identified early EPR adopters as predominantly highly educated males with chronic diseases and a regular general practitioner. Despite moderate satisfaction with the current implementation, most users recommend the system to others, suggesting a belief in its potential value. Important questions are raised regarding EPR accessibility, limited adoption by professionals, and potential digital health disparities in the general population.

## Introduction

The digitalization of health care represents a significant public health development worldwide, with electronic patient records (EPRs) emerging as a cornerstone of this transformation. EPRs are introduced to encourage patient self-management and responsibility for their own health while potentially improving care coordination and health care delivery [1,2]. Their promise to enhance health care quality, reduce costs, and improve patient outcomes has driven widespread implementation efforts across health care systems globally.

In Switzerland, the national EPR aligns with the Health2030 strategy and the Federal Law on Electronic Patient Record (LDEP [3]), which aims to establish a standardized framework for digital health records. The case of Switzerland, a confederation of 26 cantons, is particularly interesting, as it is a small country at the heart of Europe with a decentralized health care system. Its constitution signed in 1848 defines the political foundations and the principle of subsidiarity, granting specific power to the federal state, whereas 26 cantons continue to be sovereign based on their own cantonal constitution [4]. The Federal Assembly is the supreme authority of the Confederation and represents both the cantons and the people [4]. The cornerstone of the Swiss health care system is the Health Insurance Act (LaMal [5]), a federal law from 1994. However, distributions of roles and responsibilities at federal or cantonal levels are not clearly established [4]. Several limitations of the current governance structure of the Swiss health care system have been identified: health promotion initiatives not receiving sufficient support and funding, some cantons being too small to provide required health care services to their population, and the deployment of EPR to all citizens being challenging [6]. It is the latter that is the topic of this article.

In 2018, the CARA association was established to provide EPR services for patients and professionals across 5 French-speaking cantons: Vaud, Valais, Fribourg, Jura, and Geneva [7]. This platform offers both EPRs and document transfer services. By June 2025, the health platform CARA had registered approximately 32,350 records and more than 2840 health care providers and institutions.

Different terminology exists regarding electronic health and medical records: electronic health records (EHRs), electronic medical records (EMRs), and EPRs, sometimes also called patient health records. EMRs are considered an internal organizational system, while EHRs are for collaboration across institutions [8]. EMRs and EHRs are controlled by professionals, while EPRs are managed by the patient, giving the patient the authority to integrate multiple sources and control access [9]. The Competence and Coordination Centre of the Confederation eHealth Suisse oversees the coordination, development, and dissemination of information regarding the EPR, whereas the cantons are responsible for its implementation.

The EPR typically contains discharge letters, medical reports, surgical reports, laboratory results, imaging reports (radiology), prescriptions, medication lists, and vaccination data [7]. Health care providers who might contribute data include hospitals, birth centers, nursing homes, clinical laboratories, general practitioners (GPs) and specialized physicians, pharmacies, home care agencies, and therapeutic practitioners [7]. The EPR content is partial rather than comprehensive: only information deemed useful for continuity of care is published, with each health care provider determining what qualifies as relevant. As patients retain complete control over which providers can access their records and which documents are deposited, information might not flow as easily as in EHRs. While patient portals are extensions of EHRs (eg, Epic MyChart) and thus controlled by health professionals, patients oversee EPRs, deciding who has access to documents.

However, EPR adoption rates vary considerably across European countries. While Finland and Estonia have demonstrated remarkable success in implementation, countries such as France, Austria, and Germany have experienced significantly lower adoption rates [10-14]. These disparities can be attributed to differences in implementation models, with some countries opting for a single centralized database (eg, Estonia), while others have adopted multiple centralized bodies (eg, Germany) or decentralized approaches (eg, Austria) [15]. Furthermore, differences exist regarding data sharing. While the Swiss EPR is restricted to users and their health professionals to whom they have given explicit access, other countries share data with the national health insurance (eg, France) or between providers using a centralized national platform (eg, Estonia). Whether these contextual differences impact EPR use and how adoption rates would play out in a decentralized health care system, such as Switzerland, is still unknown.

The scientific literature suggests that EPRs might have a positive influence on care coordination, management, and patient safety [16]. Studies have shown that patients with access to their health records tend to develop a better understanding of their health conditions, demonstrate improved medication adherence, and experience enhanced patient-provider relationships. Furthermore, EPR access seems to encourage greater patient self-management and engagement in health care decision-making [17].

Previous research has characterized early EPR adopters as individuals with specific demographic and health profiles. These include persons with one or more chronic diseases [18], predominantly female users [19-21], and those with higher socioeconomic status and health literacy [22,23]. However, to date, no Swiss study has comprehensively examined the characteristics and experiences of EPR users within the Swiss healthcare context.

This research gap is particularly relevant given Switzerland’s fragmented health care system and its nonalignment with European health policies, characterized by cantonal autonomy in health policy implementation and a strong emphasis on patient privacy. Understanding the profiles, motivations, and experiences of EPR users and nonusers in Switzerland is crucial for informing effective implementation strategies and addressing potential barriers to adoption.

This study aimed (1) to document patients’ use of EPR in the Swiss canton of Vaud and to examine the users’ profiles; and (2) to explore the users’ and nonusers’ perceptions regarding EPR and its benefits and barriers.

## Methods

### Overview

An explanatory sequential mixed methods study was conducted in 2 phases: first collecting and analyzing quantitative data, followed by qualitative data collection and analysis [24]. A mixed methods research study has the advantage of being able to elucidate complex issues [25], and using this design, researchers can combine the strengths of 2 approaches and thereby contribute to interdisciplinary and collaborative research evidence [26]. In our study, 2 methods (quantitative questionnaire and interviews) were organized sequentially [27] and analyzed, in accordance with the rules of each method [28].

### Quantitative Study

**Figure 1.**
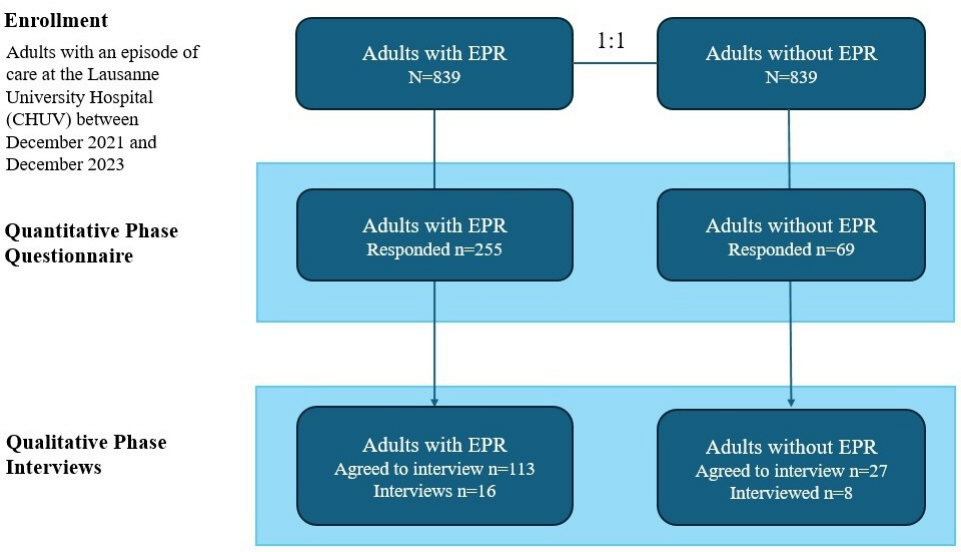
CONSORT (Consolidated Standards of Reporting Trials) flow diagram. EPR: electronic patient record.

Adult patients residing in the canton of Vaud, Switzerland, who had an episode of care (inpatient or outpatient) between December 1, 2021, and December 28, 2023, at Lausanne University Hospital (CHUV) with an active EPR were identified (N=839) and matched 1:1 with adult patients without EPR on age, self-identified gender, and care period (N=839); refer to Figure 1. Whether an EPR was present at admission was automatically verified and reconciled against the institutional EHR (CHUV).

A questionnaire (Multimedia Appendix 1) was sent to them, which they returned by post or electronically. All data were collected and managed using REDCap (Research Electronic Data Capture) hosted at CHUV [29]. Demographic data and socioeconomic profiles were collected. A high level of education was equivalent to a higher or specialized school or university. In addition, the level of health literacy and digital health literacy was assessed using a validated questionnaire (HLS19-Q12-CH [Health Literacy Survey 2019-2021] and HLS19-DIGI-CH [Digital Health Literacy], scales in French [30,31]), comparing the profile of this population to a sample of the Swiss population recently studied in 2019‐2021 (Health Literacy Survey Schweiz 2019‐2021 [31]). Voluntary participants provided their contact details for the qualitative phase (Phase 2) at the end of the questionnaire.

On the statistical level, bivariate analyses were performed using 2-tailed Student *t* test or ANOVA for continuous data, and the chi-square test for categorical data. Bivariate associations were assessed by Spearman correlation. Statistical significance was considered for a 2-sided test with *P*<.05. Means are expressed with SD. All statistical analyses were performed using Stata version 18 software (StataCorp LLC).

### Qualitative Study

The qualitative analysis was performed to understand the users’ and nonusers’ experiences and perceptions toward the EPR. Semistructured interviews with EPR users and nonusers made it possible to understand the facilitators and barriers as well as the benefits of the EPR, through thematic analysis. The recruitment was structured as follows: participants from Phase 1 provided their contact details at the end of the questionnaire if they were willing to participate in interviews. A list of 140 potential interviewees who responded positively to the invitation (113 EPR users and 27 nonusers) was generated and stratified according to age, self-identified gender, professional status, comorbidities, and health literacy scores. For the user group, a priority list was established to recruit at least 15 participants, both men and women with diverse profiles. The group of nonusers was all invited, but only 8 participants were willing to schedule an interview. The goal was to recruit 20-25 interviewees with diverse profiles among participants, so that a range of perceptions could be collected.

### Interviews: Data Collection and Analysis

Participants shared their perceptions, their engagement in that experience, and evaluated their experiences regarding the EPR. An interview guide was developed (Multimedia Appendix 2), and pilot interviews were conducted to assess the suitability of the questions. The duration of interviews ranged from 15 minutes to 1 hour 12 minutes (mean 39, SD 17 minutes). To ensure the transparency and validity of the findings, all interviews were conducted in accordance with the interview guide. The interviews were transcribed “ad verbatim” in French (except 1 interview conducted in English) and data were analyzed using thematic analysis [32] with the help of MAXQDA version 24 (VERBI GmbH).

The 6 steps described by Braun and Clarke [32] were used to transcribe the data (step 1), followed by systematically coding the collected data (step 2). The next phase included a compilation of codes into themes (step 3) that were then reviewed (step 4) and discussed. Three researchers (VS, MR, and CL) worked together on the dataset, discussing themes, and refining the relations between the different themes (step 5) and writing it all up (step 6). An Excel table allowed for transparency of the process, being able to compare themes, subthemes, and citations. It must be noted that the reflexive thematic analysis of Braun and Clarke [33], rather than using a codebook approach or a coding reliability approach based on a positivist paradigm [34], was in line with the interpretative analysis of the data.

While the entire data analysis process was performed in French, for this article, themes, subthemes, and citations are translated into English.

### Data Integration

At first, data from phases 1 and 2 were analyzed separately and integrated during the interpretation phase to find explanations of the different uses and perceptions of EPR. The integration of the quantitative and qualitative phases happened at 2 levels: first, based on the quantitative results, the selected participants with varied characteristics ensured a rich representation of responders. This approach allowed us to link qualitative and quantitative data at the individual level, and an in-depth analysis of this subgroup was performed. Second, a follow-up analysis was performed based on the quantitative data from Phase 1 [27] to see whether qualitative data were linkable to results from the quantitative data at the aggregate level.

Interpretation was all done in the original language (French), and translation of the quotes into English was performed only for the purpose of authoring this paper.

### Reflexivity

The concept of reflexivity was adopted throughout the study to ensure its scientific rigor [35]. Specifically, reflexivity is defined as a thoughtful and conscious process that includes continuous evaluation of subjective responses, interpersonal dynamics, and the research process itself [36]. Discussions among the research group members from different disciplinary backgrounds (medicine, physiotherapy, and sociology) facilitated the triangulation of perspectives.

### Ethical Considerations

This mixed methods study was conducted in accordance with the Declaration of Helsinki and received ethical approval from the local ethics committee (Commission cantonale d’éthique de la recherche sur l’être humain, CER-VD, no 2023‐01672). Participation was voluntary and all participants were fully informed about the study’s objectives, procedures, and their right to withdraw at any time without consequences. Written informed consent was obtained from all participants prior to completing the questionnaire (Phase 1). For Phase 2, additional informed consent was obtained from the subset of participants selected for in-depth interviews. No compensation was provided. Participant anonymity was maintained throughout the study. All questionnaire responses and interview data were deidentified and assigned unique identification codes. Confidentiality was ensured through secure data storage with password-protected access limited to the research team members.

## Results

### Phase 1: Quantitative Study  

#### Overview

The overall participation rate in this phase was 19.3% (324/1678), with a marked difference between EPR users (255/839, 30.4%) and nonusers (69/839, 8.2%). Men were overrepresented both in the initial population with an EPR (508/839, 60.5%) and among the study participants (185/255, 72.5%), as shown in Table 1. The low participation of nonusers is a methodological limitation. Nonusers were more difficult to recruit, possibly due to a lack of interest or knowledge about the EPR.

Patients who have opened an EPR tended to have a higher education, defined as higher or specialized school, or university (155/255, 60.8%). They are very often affected by chronic illnesses (204/255, 80%) and have access to an extensive health care network: 201/254 (79.1%) of them consulted 2 or more health care professionals in the last 3 months. Most of this population (249/253, 98.4%) had a GP. Patients with EPRs did not visit the emergency departments less frequently during the last 3 months (60/254, 23.6%). Refer to Table 1.

**Table 1.** Sociodemographic characteristics, health status, and use of health services of participants according to their EPR^a^ adoption status (n=324).

Characteristics	With EPR	Without EPR	*P* value
Self-identified gender, n/N (%)			.04
Men	185/255 (72.5)	41/69 (59.4)	
Women	70/255 (27.5)	28/69 (40.6)	
Age (years), mean (SD)	60.4 (14.6)	60 (14.5)	.81
Married, n/N (%)	147/253 (58.1)	38/68 (55.9)	.87
Lives alone, n/N (%)	58/255 (22.8)	19/67 (28.4)	.77
Higher education, n/N (%)^b^	155/255 (60.8)	34/69 (49.3)	.09
General practitioner, n/N (%)	249/253 (98.4)	64/68 (94.1)	.04
Poor or very poor health, n/N (%)	29/254 (11.4)	8/69 (11.6)	.97
With chronic illness, n/N (%)	204/255 (80)	50/68 (73.5)	.10
Two or more health care providers during the last 3 months, n/N (%)	201/254 (79.1)	47/68 (69.1)	.047
One or more emergency department visits during the last 3 months, n/N (%)	60/254 (23.6)	12/68 (17.7)	.29

a EPR: electronic patient record.

b Higher education is defined as University or University of Applied Sciences (HES).

#### Use of and Satisfaction With EPR

Of the 251 participants with an EPR, 96 (38.3%) reported using it once during the last quarter, and 94 (37.5%) reported using it 2 or more times. Among EPR users, 76/252 (30.2%) stated that they were satisfied with the EPR; the reasons reported for this moderate level of satisfaction are essentially linked to the themes of poor compliance by private practice physicians (GPs), other hospitals and clinics outside the university hospital, or the difficulty in obtaining medical documents. The complexity of use and the lack of a user-friendly platform were also cited. While no correlation was observed between self-identified gender and satisfaction levels, educational attainment was strongly associated with satisfaction levels. Participants with higher education levels were significantly more critical, demonstrating a dissatisfaction rate of 98/155 (63.2%) compared to 40/97 (41.2%) among those with lower educational levels (*P*=.001). In contrast, no significant correlations were identified with either health literacy or digital health literacy levels.

Despite these reported limitations, a substantial majority of users (172/254, 67.7%) indicated they would recommend opening an EPR to someone close to them.

#### Relationship With Medical and Health Professionals

The impact of the EPR on the physician-patient relationship is positive or neutral overall. A total of 33.1% (84/254) of users think that the EPR has an impact on the relationship with the GP; this is most often reported as unchanged (49/79, 62%), often improved or much improved (26/79, 32.9%), or rarely deteriorated (4/79, 5.1%).

#### EPR Use and Anxiety

The effect on anxiety remains marginal. Eleven users (11/252, 4.4%) reported an effect on the level of anxiety; this proportion is comparable with other studies [37,38]. Only 1 participant reported that their anxiety had worsened through EPR use.

#### Health Literacy

Participants with EPR tended to have a higher health literacy score (questionnaire HLS19-Q12-CH) with a mean score of 83.5 (SD 18.8) than those without EPR (mean 81.5, SD 23.8), although this difference was not statistically significant (*P*=.99). By comparison, the Swiss Health Literacy study (HLS19-21-CH) [31] reported an average score of 77.3 (SD 19.6) at the national level, and 80.2 for the canton of Vaud, which ranks first among Swiss cantons. Early adopters of the EPR thus stand out for having a higher level of health literacy, which may be linked to the level of education reported in the questionnaire.

**Figure 2.**
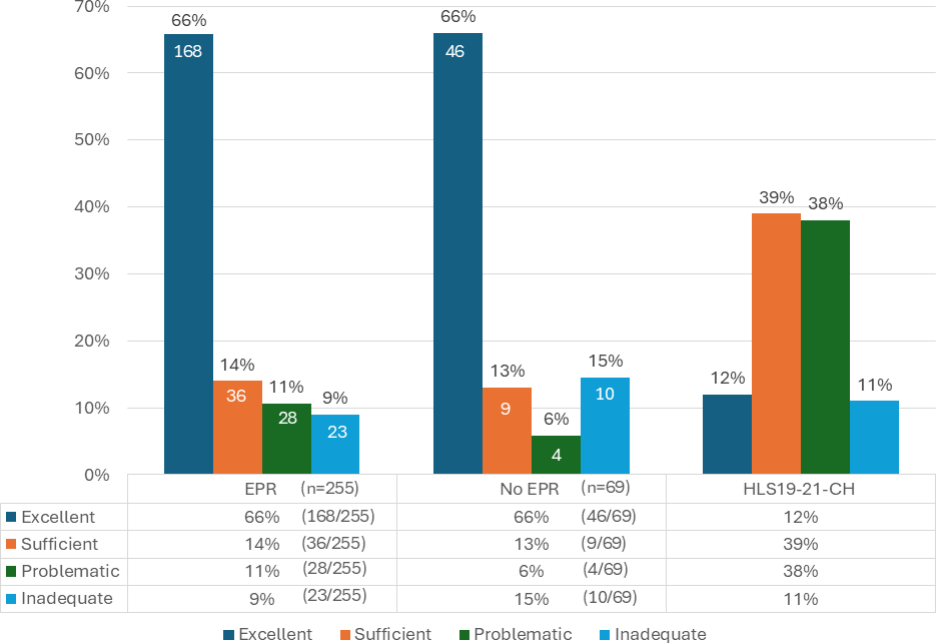
Distribution of health literacy levels (HLS19-Q12) by electronic patient record adoption status: comparison with the Swiss study [31]. EPR: electronic patient record; HLS19-21-CH: Health Literacy Survey 19-21.

According to the definitions of the M-POHL 2023 categories [30], two-thirds of patients with or without an EPR (168/255, 65.9% vs 46/69, 66.7%, respectively) are considered to have an excellent level of health literacy; 51 EPR users (51/255, 20%) have a level of literacy considered insufficient (ie, problematic or inadequate). Among nonusers, the proportion of patients with an insufficient level is the same (14/69, 20.3%), including 10/69 (14.5%) with an inadequate level. By way of comparison and considering a change in the definition of the categories since 2023, the population with EPRs in our study has better health literacy than the general population [31], with a proportion of 49% (1226/2502) of the Swiss population reporting frequent difficulties in processing health information, linked to an inadequate level of literacy (Figure 2).

#### Digital Health Literacy

Patients with EPRs tended to have a slightly higher level of digital literacy (mean 60.4, SD 31.5), although this difference was not significant compared with the group without EPRs (mean 59.1, SD 36.0; *P*=.92). This population also tended to interact more with digital resources in the health context (mean 74.5, SD 35.5 vs mean 63.5, SD 43.5; *P*=.11), which is logically reflected in the regular use of the EPR, included in this category.

### Phase 2: Interviews With EPR Users and Nonusers

#### Overview

For the qualitative interview study, we included 16 users and 8 nonusers of the EPR, totaling 24 participants (Table 2). While we were able to recruit an equal number of men (n=11) and women (n=13), it was more difficult to recruit EPR nonusers (n=8) compared to EPR users (n=16). It has also been noted that there is an overrepresentation of participants with a university degree (15 participants with a university degree and 2 with a postsecondary education) compared to a secondary education (high school level). Furthermore, retired persons made 50% (12/24) of the interviewed population with a slight overrepresentation of women (5 men and 7 women).

**Table 2.** Population interviewed (n=24).

ID	Gender (self-identified)	EPR^a^ (yes or no)	Highest degree	Current professional activity
PERS1	Man	Yes	University	Retired
PERS2	Man	Yes	University	Retired
PERS3	Man	Yes	University	Retired
PERS4	Woman	No	Not known	Retired
PERS5	Woman	Yes	Not known	Retired
PERS6	Woman	Yes	Secondary education	Employed
PERS7	Woman	No	University	Manager
PERS8	Man	Yes	University	Manager
PERS9	Woman	Yes	University	Manager
PERS10	Woman	Yes	Higher postsecondary education (not university)	Employed
PERS11	Woman	Yes	University	Employed
PERS12	Woman	No	University	Manager
PERS13	Man	Yes	Higher postsecondary education	Not currently in the workforce
PERS14	Woman	Yes	Higher postsecondary education	Retired
PERS15	Woman	No	University	Retired
PERS16	Man	Yes	University	Retired
PERS17	Man	No	University	Manager
PERS18	Woman	Yes	University	Retired
PERS19	Woman	Yes	University	Retired
PERS20	Woman	No	Secondary education	Retired
PERS21	Man	No	University	Employed
PERS22	Man	Yes	University	Retired
PERS23	Man	No	Secondary education	Self-employed
PERS24	Man	Yes	Not known	Not currently in the workforce
Total	13 women and 11 men	16 persons with EPR and 8 persons without EPR		

a EPR: electronic patient record.

Six themes were identified during the thematic data analysis which are presented in detail below: digitalization, the Swiss context of the EPR, perceptions of the EPR, EPR as a tool, health literacy, and communication. To illustrate and make explicit the different themes, subthemes, and selected quotes, please refer to Table 3.

Most early adopters wanted to open an EPR because they were interested in tracking their health data and taking an active role in their own health, since many of them have chronic illnesses. Second, the desire to facilitate coordination between health care professionals to achieve optimal health care is also found among nonusers. The EPR context in Switzerland hampers its optimal use, as it is currently not compulsory for Swiss citizens (opt-in strategy). Among health care providers, only hospitals, birth centers, nursing homes, and physicians granted practice rights since 2022 are required to participate in the documentation and use of the EPR. The impact of this is the very low level of support and appreciation that early adopters encounter from professionals.

**Table 3.** Themes, subthemes, and quotes to illustrate findings.

Theme	Subtheme	Example quotes
Digitalization	Enablers of digitalization	“Yeah, quite a bit. Actually, I'm proud of not having to carry a wallet. I do everything with my watch. My children also encourage me a bit.” (PERS11, User)
	Limitations of digitalization	“So I imagine that's what will naturally scare a lot of people, it's this data, naturally.” (PERS24, User)
	Level of knowledge	“At home, I have everything electronic, so I mean all the files, all that, encrypted, of course.” (PERS1, User)
	Optimized usage of IT tools	“I also think that information provides security; the more information we have, the safer we feel.” (PERS11, User)
	Artificial intelligence	“And I'm also quite interested in everything related to artificial intelligence.” (PERS17, Non-user)
	Advanced state of digitalization	“There's the security aspect, of course, but I trust it.” (PERS11, User)
Swiss context	Level of knowledge of the EPR^a^ among the Swiss population	“Given the number of members, I don't think so. I get the impression that people think it's something for geeks, or at best, maybe they think it was set up by doctors. And then it's something between them. When I talk about it a little, no one knows anything about it.” (PERS22, user)
	EPR requirement	“I think it should simply be mandatory.” (PERS8, user)
	Level of patient engagement	“I don't see how we can convince patients to make this effort (...). I mean, if they don't use it, there's no point. I don't need it, you know? I have everything on paper. I've made my own file at home with everything that's happened to me.” (PERS19, user)
	Level of health care professional engagement	“Now I think the implementation and the problem is that doctors don't use it. So there you go... it's useless.” (PERS19, user)
	Nonmandatory affiliation with the EPR	“If you have an identity that is truly nationally mandated. But anyway, we need it, we also need it to vote digitally. One day it will come, it's quite reliable.” PERS1, user)
	Lack of standardization	“There are as many EPRs as there are organizations. Each one has developed its own.” (PERS3, user)
	Power of insurers	“But it's true, when you think about it, I don't want my insurance to be able to keep up. Then I say to myself: No one has time to check if [PERS4] weighs three pounds more. But maybe they do, so they can send me dietary restrictions.” (PERS4, non-user)
Communication	Communication: between peers	“Because if we say: The doctor at the hospital where I arrive in an emergency can know everything about me, it's a win-win situation.” (PERS4, non-user)
	Communication: patient-professional	"So in my opinion, if the electronic health record becomes standard, it increases transparency between the patient and the doctor." (PERS1, user)
	Professional barriers to the EPR	“Because we know they're all already overloaded, even the assistants, secretaries, and so on. It's a disaster. ” (PERS13, user)
Health Literacy	Information seeking	“Especially on the internet because there's too much fake stuff, even if you find multiple sources. And then you often compare them, and they just copy each other. So to know what's really reliable, you can't trust it.” (PERS1, user)
	Level of engagement in personal health	“Also, taking on more responsibility in the healing and treatment process. Not delegating everything related to health to the health insurance company and the demigods in white coats, right?” (PERS11, user)
EPR as a tool	Opening of the EPR	"The registration process was too complex, but I think it has improved." (PERS22, user)
	Content	"Well, maybe it's my fault, but I can't get people to put my files in my dossier." (PERS5, user)
	Development proposals	“So, this is a case I should also tell you about, but I created some documents at [community hospital]. The document is scanned in a certain format, then the [University Hospital] receives it and processes it in a different format. Different departments at the hospital do it with completely different titles and things like that. There's nothing organized or standardized, so we were completely lost; it's a disaster.” (PERS22, user)
	Perceptions of the EPR	“So, if I wanted to get into this now, I would also like to have some support. Exactly. Because me, all alone at home typing in Swisspass, I don't know what. I can get to the train website anywhere, but I don't know if I'm really getting to the right place.” (PERS4, non-user)
	Use of EPR	“I think that access should be given to all professionals. But to what extent? Healthcare professionals, for example, doctors, we're talking about nurses. I might disagree with a nurse I don't know who has never seen me but who has seen my name? I don't know, on some document. And then who looks at my file, I wouldn't like that. But a doctor, it should stop at the doctor's level.” (PERS21, non-user)

a EPR: electronic patient record.

#### Digitalization

The approach to digitization is perceived differently, with some contrasting it with the loss of human contact, while others see it as a valuable aid. Some participants are very advanced in their use of digitalization and technology and use this technology to gather data:

So, I use an app that’s connected to the device that measures blood pressure. Oh yeah, arterial. That’s what I’ve been doing for a while. But it’s to keep track of it. And then I have all the values on my phone, and I can show them to a doctor, to my GP. When I go to see him, yes, I’ll show him all the measurements I’ve taken over the last few months.

[PERS19]

I have a little app that reminds me when I need to take medicine to make sure I take it and I don't forget.

[PERS23]

It is observed that older participants interviewed were also connected, not only the younger population:

Yeah, enough of that. I mean, I’m proud of the fact that I don’t carry around a wallet. I do everything with my watch. It’s also my kids who push me a bit.

[PERS11]

Overall, interviewees were quite at ease with digital tools, as can also be seen in the quantitative data with statistically nonsignificant differences in scores of digital health literacy.

#### Swiss Context of the EPR

The Swiss context in which the EPR has emerged has a substantial impact on the level of uptake. Participants have a negative perception of the lack of standardization and the multitude of digital health tools in Switzerland. They feel misinformed. In fact, many EPRs in French-speaking Switzerland have slightly different objectives and were not interoperable at the time of the interviews.

For participants, the noninvolvement of health care professionals, particularly GPs, is a significant obstacle to their use of EPR:

Ah well, doctors would have to use it first. If doctors don’t use it. I don’t see how we can convince patients to make the effort, ie, they’re the ones holding the knife by the handle. I mean, if they don’t use it. There’s no, no reason for it. I mean, I don’t need it. For me, I’ve got everything on paper. I’ve made my own file which is at home with everything that’s happened to me.

[PERS19]

As currently not compulsory for Swiss citizens, early adopters perceive that registration with the EPR has a negative impact on the low level of support from professionals.

I think it should be compulsory for everyone to work with it. Otherwise, it won’t work if a doctor doesn’t, if a physiotherapist doesn’t want to, it really should be a given that everyone on this platform, after of course the data protection issues for the mass of data protected but, there are solutions.

[PERS11]

Some participants proposed that EPR be assigned to every citizen at birth.

#### Perceptions of the EPR

The EPR is perceived positively by all participants, even those who were not users of the tool. They cite the benefits of limiting health care costs by reducing duplicate examinations and limiting errors in care. For patients, the EPR helps facilitate collaboration between health care professionals, ultimately leading to improved care.

The motivations of early adopters included having an interest in tracking their health data and taking an active role in their own health, since most of them have chronic illnesses. Second, they aimed to facilitate coordination among health care professionals, with the goal of achieving optimal health care management.

#### EPR as a Tool

Everyone agrees that the EPR tool was not designed according to the patients’ needs. Participants registered with the EPR perceive numerous barriers to its optimal use: difficulties linked to the high security level of the platform (multifactor authentication and no mobile app), classification by date in order of insertion, and difficulty in searching for documents. As a result of the interviews, participants from both groups concurred that the platform needs to be enhanced to improve its user-friendliness. Table 4 presents participants’ suggestions for improving the platform.

**Table 4.** Suggestions for improvement proposed by participants.

Suggestions	Description
Access to EPR^a^	Easier access to the platform, by using a mobile app and equalizing access to all health care professionals.
Data centralization	To reduce redundancy of tests (lab and imaging), available updated medication list and improved health professional coordination.
Search function	To be able to easily find historical data on the platform.
Information	Health professionals can upload personalized and reliable information (including comorbidities, medication list, exercise videos, contraindications, precautions, and allergies) for self-care.
Calendar and alerts	Appointment reminders, vaccine boosters, or screening tests out of date.
Network	List of all providers used by patients including specialists, therapists, dentists, osteopaths, chiropractors, paramedical providers such as optometry, podiatry, pharmacy, social workers, dietetics, and complementary services, family members, and emergency contacts.
Dashboard	Graphical representation of essential health care data such as vital signs, labs, pain scale, Functional Independence Measure, BMI, and hemoglobin A_1c_.
Advanced directives	Information about health care power of attorney and medical decisions, and other relevant legal implications.

a EPR: electronic patient record.

#### Health Literacy

The early adopters were already very committed to their health, archiving their health data at home and taking a proactive stance in their health.

Afterwards, I've always been someone who likes to have a look at my treatment, who wants to understand why things are done and why they're not done differently. So, I've always asked my doctors a lot of questions, either by e-mail, or in person, in consultation with them, or by telephone. So, I've always taken a lot of notes from people, a lot of letters. In fact, there’s a lot of information that I got, but because I led a process, in quotation marks, of proactive research to get this information, it wasn't something that was easily accessible. So, in a way, I had the advantage of already being well informed.

[PERS24]

There were also some questions about the level of trust in the use of the internet in seeking health information. Health care professionals (especially doctors) have remained a trustworthy source of information.

#### Communication

For EPR users, having access to their medical data makes them feel more comfortable communicating with health care professionals and moving toward a more transparent relationship, reducing the divide between medical doctors and patients.

The interaction between patient and professional is more on an equal footing. Today it’s a black box, but having access to the data allows me to have confidence.

[PERS2]

And so in my opinion, if the electronic record becomes standard, it increases transparency between the patient and the doctor.

[PERS1]

It has been noted that the doctor-patient relationship is changing, yet it does not necessarily give more power to the patient:

I don’t think the patient is really given more importance. They have more information, they have more knowledge. I can’t say no, I don’t take this medicine without listening, he suggested it to me because I think that with this information, I can’t be the doctor. Yes, I think patients should interpret this as a more comprehensive source of information.

[PERS23]

As this relationship evolves, patients see the use of the EPR as a means of control, encouraging greater transparency between professionals and patients.

Doctors are much more challenged than they used to be, aren't they? People are looking for much more.

[PERS17]

The data analysis identified a second point related to communication, which is removing the barrier to patients’ understanding of certain technical terms and professional jargon. Being able to read and look up specific terms can improve patients’ overall understanding of their health (health literacy).

Yeah, that’s the jargon that’s used. Or else we don’t take much time to explain it to you because we’re pretty strict about our schedules. So if you don’t have a basic understanding, you haven’t retained anything, basically.

[PERS 22]

In summary, with the use of the EPR, communication between patients and professionals could be more transparent. However, for the interviewees, the concern is not necessarily related to the patient-professional relationship, but rather with interprofessional communication, which seems much more relevant.

## Discussion

### Principal Findings

This mixed methods study provides valuable insights into the characteristics, experiences, and perceptions of EPR users and nonusers in the canton of Vaud, Switzerland. Our findings reveal that early adopters of EPRs in Switzerland demonstrate distinct demographic and health profiles that both align with and diverge from patterns observed in previous international research.

### Characteristics of Early Adopters

Our results indicate that early EPR adopters in French-speaking Switzerland are predominantly characterized by higher educational levels, chronic health conditions that require regular monitoring, and extensive health care networks. These individuals typically demonstrate higher levels of health literacy and were already actively engaged in managing their health information prior to the adoption of EPR, often through personal archiving systems. These findings align with previous studies that have identified chronic disease status as a significant predictor of EPR adoption [11,18].

However, contrary to international trends that typically show higher female participation in EPR adoption [19-21], our study found a significant male predominance among Swiss early adopters. This gender disparity may reflect broader patterns of digital technology engagement in Switzerland, where technical and IT fields continue to show marked gender disparities characterized by predominant male representation. This unexpected finding warrants further investigation to understand the sociocultural factors influencing gender-based differences in EPR adoption within the Swiss context.

### Health Literacy and Digital Engagement

Our study reveals that early EPR adopters demonstrate higher levels of health literacy compared to both nonusers and the general Swiss population. This finding is consistent with previous research that highlights the relationship between health literacy and the adoption of digital health tools [22,23]. The proactive health information-seeking behaviors observed among early adopters suggest that these individuals perceive themselves as legitimate partners in patient-professional relationships, potentially transforming traditional hierarchical dynamics toward more horizontal communication patterns. Similar transformations have been observed in pediatric settings, where parents of chronically ill children demonstrate comparable engagement patterns [39].

### Barriers to Adoption and Implementation

Despite recognizing potential benefits, both users and nonusers identified significant barriers to the optimal implementation of EPR. These include technical challenges (such as security procedures and interface design), limited professional participation, and fragmented implementation across the health care system. The critical role of health care professionals as facilitators or barriers to patient EPR adoption emerged as a consistent theme, with many participants perceiving negative attitudes toward EPRs among health care providers. This finding aligns with observations from France and Austria, where professional resistance has similarly hindered EPR implementation [40,41].

Our qualitative findings also revealed concerns about illness stigmatization influencing EPR adoption decisions, particularly for sensitive health conditions. Some participants expressed reluctance to include mental health or gynecological information in their EPRs due to perceived stigma. This observation aligns with German research indicating that patients with stigmatized conditions demonstrate greater resistance to EPR adoption [42] and Norwegian findings showing more critical attitudes toward EPRs among patients with mental health conditions compared to those with physical disorders [43].

### System Design and User Experience

The relatively low satisfaction rates among EPR users (30.2%) primarily stem from limitations in system design and implementation rather than the concept itself. EPR users expressed disappointment with the current EPR tool’s functionality, citing a lack of intuitive design, poor document classification, and limited portability. These findings align with global experiences in EPR implementation, where user experience significantly impacts adoption rates and perceived effectiveness [39]. This misalignment of expectations represents a significant challenge for successful implementation.

### Contextual Factors Influencing Adoption

Our study highlights the importance of considering sociopolitical contexts in EPR implementation. The Swiss opt-in approach to EPR registration, which makes adoption voluntary for citizens but mandatory for newly established health care providers since 2022, has created an implementation gap. This policy approach contrasts with opt-out strategies implemented in countries such as Austria, which have demonstrated different adoption patterns [40]. The influence of professional associations, particularly the perceived resistance from medical societies, emerged as a significant barrier to widespread adoption.

Additionally, our findings suggest that individual factors such as polymorbidity, emotional state, and social support networks influence EPR adoption decisions, consistent with Austrian research demonstrating correlations between these factors and EPR adherence [44].

### Implications and Future Directions

Our findings have significant implications for the implementation of EPR in Switzerland and similar health care contexts. First, the distinct profile of early adopters suggests that current implementation approaches may be inadvertently creating digital health disparities, with higher-educated, male, chronically ill patients benefiting disproportionately. Targeted strategies to engage underrepresented populations, particularly those with lower health literacy and fewer digital skills, are essential for equitable implementation.

Second, the critical role of health care professionals in facilitating or hindering EPR adoption highlights the need for comprehensive professional engagement strategies. Future implementation efforts to foster greater support for EPR adoption should address professional concerns regarding workflow integration, information accuracy, and time constraints.

Third, the technical and design limitations identified by users suggest the need for user-centered redesign processes that incorporate feedback from diverse stakeholders. Improvements in interface design, document classification, and mobile accessibility could significantly enhance user satisfaction and adoption rates. Participants suggested a wide array of suggestions for improvement (Table 4), useful for policymakers and organizations responsible for implementation. Easier access to the platform (mobile app) and better organization and standardization of documents are needed to respond to the clients’ needs.

Future research should explore the gender disparity observed in our study, investigate effective strategies for engaging underrepresented populations, and evaluate the impact of different policy approaches (opt-in vs opt-out) on EPR adoption patterns in the Swiss context.

### Limitations

Several limitations should be considered when interpreting our findings. The significant gender imbalance in our sample, with male participants substantially overrepresented among both EPR users and study respondents, introduces potential systematic bias. Similarly, the low response rate among nonusers (8.2% compared to 30.4% among users) may have resulted in selection bias, potentially overrepresenting individuals with stronger opinions about EPRs or higher digital literacy. Furthermore, it would have been very interesting to compare respondents to nonrespondents to better understand the population of nonrespondents. However, due to restrictions by the ethics committee and data availability (eg, socioeconomic factors, comorbidities, and literacy), we could not explore these dimensions.

Furthermore, investigating health literacy and digital health literacy using standardized questionnaires (HLS19-Q12 and HLS19-DIGI-CH) can be criticized. A recent scoping review [45] identified 33 studies using digital health literacy tools; however, with several limitations, such as “narrow scope, limited adaptability to the rapidly evolving digital landscape, and lack of objective testing” (p. 11). The choice of the 2 instruments used in this study was based on the possibility to compare them to results from the national survey [31]. This choice aligns well with Thorup et al [45] recommendations to (1) combine health literacy and digital health literacy (multidimensional tool), (2) to use a validated tool, and (3) to use tools that are context specific.

Our qualitative analysis, while providing rich contextual insights, is inherently subjective and may reflect researchers’ interpretations. Despite efforts to ensure analytical rigor through team-based analysis and reflexive practices, alternative interpretations of the data remain possible.

Finally, our study focused on patients with care episodes at a university hospital, which may limit its generalizability to community-based settings or rural populations. The experiences and perceptions of individuals without recent hospital encounters may differ substantially from those captured in our sample.

### Conclusion

This study provides valuable insights into the characteristics, experiences, and perceptions of early EPR adopters in the canton of Vaud, Switzerland. Our findings reveal that these early adopters constitute a distinct population characterized by higher education levels, chronic health conditions, extensive health care networks, and higher health literacy. Despite moderate satisfaction with the current EPR implementation, most users recommend the system to others, suggesting belief in its potential value despite existing limitations.

The observed demographic patterns raise important questions about EPR accessibility and potential digital health disparities. The significant male predominance among Swiss early adopters contrasts with international trends and warrants further investigation. Additionally, the higher health literacy levels among adopters suggest that current implementation approaches may inadvertently favor already advantaged populations.

Our mixed methods approach revealed that moderate satisfaction among users primarily stems from limited system uptake across the health care ecosystem rather than inherent opposition to the concept. The optimal effectiveness of EPRs appears contingent upon systemic integration throughout the ambulatory care network, suggesting that extending implementation beyond university hospitals is essential for achieving the intended goals of care continuity and coordination.

As Switzerland continues its digital health transformation, addressing the technical, professional, and policy barriers identified in this study will be crucial for realizing the full potential of EPRs to enhance health care quality, coordination, and patient engagement.

## Supplementary material

10.2196/83702 Multimedia Appendix 1 Questionnaire translated in English.

10.2196/83702 Multimedia Appendix 2 Interview guide translated in French.

## Abbreviations

CHUV: Lausanne University Hospital
CONSORT: Consolidated Standards of Reporting Trials
EHR: electronic health record
EMR: electronic medical record
EPR: electronic patient record
GP: general practitioner
HLS19-DIGI: Digital Health Literacy Survey
HLS19-Q12: Health Literacy Survey
LDEP: the Federal Law on Electronic Patient Record
REDCap: Research Electronic Data Capture
