# Supplementary material for: Usage and Perceptions of Electronic Patient Records Experienced by Users and Nonusers in the Canton of Vaud, Switzerland: Mixed Methods Study
Source: J Med Internet Res. 2026 Apr 21;28:e83702. doi: 10.2196/83702 (PMC13107525; doi:10.2196/83702)
Supplement: Multimedia Appendix 1 [file jmir-v28-e83702-s001.pdf]

Appendix A – Questionnaire (English)

HESAV

Centre hospitalier universitaire vaudois

Questionnaire Study EPR Vaud

1. **What is today's date?**

   ☐☐. ☐☐.2024
   day month

2. **Do you currently have an Electronic Patient Record (EPR)?**

   The Electronic Patient Record (EPR) is a collection of personal documents that contain health-related information. You have taken the step of opening one, or one of your relatives (representative) has taken it for you.

   - ○ Yes
   - ○ No

Let's start with some general questions.

3. **What is your gender?**

   - ○ Male
   - ○ Women
   - ○ Non-binary

4. **What is your marital status?**

   - ○ Single
   - ○ Married
   - ○ Divorced
   - ○ Widowed or widowed
   - ○ Unmarried, bound by a registered partnership
   - ○ Partnership dissolved judicially
   - ○ Partnership dissolved by death
   - ○ Partnership dissolved thereafter by declaration of absence

5. **What is your nationality?**

Several answers are possible.

- ☐ Swiss
- ☐ French
- ☐ German
- ☐ Italian
- ☐ Portuguese
- ☐ Spanish
- ☐ Other nationalities.

If you answered "other nationalities":
Can you please specify which one(s)?

____

6. **What is your mother tongue?**

Several answers are possible.

- ☐ English
- ☐ Swiss-German
- ☐ Italian
- ☐ English
- ☐ German
- ☐ Portuguese
- ☐ Spanish
- ☐ Other

If you answered "other", can you please specify which one(s)?

____

7. **What is the situation that best describes you at the moment?**

- ○ You live alone
- ○ Single-parent family
- ○ As a couple without children
- ○ As a couple with children
- ○ Other

If you answered "other", can you please specify?

____

8. **What is the highest level of education you have achieved?**

- ○ Compulsory school (primary)
- ○ Secondary school (high school)
- ○ Apprenticeship (post-obligatory degree)
- ○ University of Applied Sciences (UAS)
- ○ University degree (University or Federal School of Technology)
- ○ PhD
- ○ Other

If you answered "other", can you please specify?

____

9. **What is your current professional activity?**

- ○ Executive or professional with graduate studies (e.g. director, executive member, lawyer, professor, engineer)
- ○ Healthcare professional (e.g. doctor, nurse)
- ○ Intermediate professional (e.g. technician, teacher, police officer, journalist)
- ○ Small Independent Professional (SME)
- ○ Employee
- ○ Worker
- ○ Unskilled worker
- ○ Apprentice or student
- ○ Unemployed person
- ○ Non-active person in (continuing) training
- ○ Non-active person in retirement
- ○ Non-active disabled person
- ○ Non-active person in a home
- ○ Other non-active person
- ○ Other

If you answered "other", can you please specify?

____

It is not always easy to obtain useful, clear and understandable information on health-related topics. Often, there may be small difficulties or problems.

We would like to know what can cause certain difficulties in the field of health.

Please indicate how easy or difficult you think the following is in each case.

10. **On a scale from very easy to very difficult, how easy would you say it is...**

10.1 **... to find out where to get professional help when you are ill?** (e.g. doctor, nurse, pharmacist or psychologist)

- O Very easy
- O Easy
- O Difficult
- O Very difficult
- O I don't know

10.2 **... to understand information about what to do in a medical emergency?**

- O Very easy
- O Easy
- O Difficult
- O Very difficult
- O I don't know

10.3 **... to judge the advantages and disadvantages of the different treatment options?**

- O Very easy
- O Easy
- O Difficult
- O Very difficult
- O I don't know

10.4 **... to act on advice from your doctor or pharmacist?**

- O Very easy
- O Easy
- O Difficult
- O Very difficult
- O I don't know

10.5 **... to find information on how to handle mental health problems?** (e.g. stress, depression or anxiety)

- O Very easy
- O Easy
- O Difficult
- O Very difficult
- O I don't know

10.6 **... to understand information on recommended screenings and examinations?** (e.g., colorectal cancer screening, blood glucose testing)

- O Very easy
- O Easy
- O Difficult
- O Very difficult
- O I don't know

10.7 **... to judge if information on unhealthy habits, such as smoking, low physical activity or drinking too much alcohol, are reliable?**

- O Very easy
- O Easy
- O Difficult
- O Very difficult
- O I don't know

10.8 **... to decide how you can protect yourself from illness using information from the mass media?** (e.g. newspapers, television or the Internet)

- O Very easy
- O Easy
- O Difficult
- O Very difficult
- O I don't know

10.9 **... to find information on healthy lifestyles such as physical exercise, healthy food or nutrition?**

- O Very easy
- O Easy
- O Difficult
- O Very difficult
- O I don't know

10.10 **... to understand advice concerning your health from family or friends?**

- O Very easy
- O Easy
- O Difficult
- O Very difficult
- O I don't know

10.11 **... to judge how your housing conditions may affect your health and well-being?**

- O Very easy
- O Easy
- O Difficult
- O Very difficult
- O I don't know

10.12 **... to make decisions to improve your health and well-being?**

- O Very easy
- O Easy
- O Difficult
- O Very difficult
- O I don't know

The next set of questions is about digital health-related information offerings. These include websites, social networks (including online forums), health applications or digital interactions with the health system/health services.

11. **In a typical week, how many days a week do you use the following digital resources to get health-related information?**

11.1 **Health-related websites**

- O Less than once a week
- O 1-3 days a week
- O 4-6 days a week
- O Once a day
- O More than once a day
- O Does not apply in my case
- O I don't know

11.2 **Social networks (including online forums) to discuss health issues**

- O Less than once a week
- O 1-3 days a week
- O 4-6 days a week
- O Once a day
- O More than once a day
- O Does not apply in my case
- O I don't know

11.3 **A digital device related to health or medical care**
(e.g. pedometer, connected watch, fitness bracelet, etc. with the exception of a mobile phone)

○ Less than once a week
○ 1-3 days a week
○ 4-6 days a week
○ Once a day
○ More than once a day
○ Does not apply in my case
○ I don't know

11.4 **Health-related apps on your mobile phone**
(e.g. for calculating calorie consumption, making it easier to take medication, measuring physical activity, improving sleep hygiene, etc.)

○ Less than once a week
○ 1-3 days a week
○ 4-6 days a week
○ Once a day
○ More than once a day
○ Does not apply in my case
○ I don't know

11.5 **Digital interactions with medical services**
(e.g., online appointment booking, access to personal medical records, electronic transmission of medical test/examination results, communication with a caregiver such as a doctor, nurse, including pharmacist, etc.)

○ Less than once a week
○ 1-3 days a week
○ 4-6 days a week
○ Once a day
○ More than once a day
○ Does not apply in my case
○ I don't know

11.6 **Other digital health-related information**

○ Less than once a week
○ 1-3 days a week
○ 4-6 days a week
○ Once a day
○ More than once a day
○ Does not apply in my case
○ I don't know

Now we'd like to know how easy or difficult it is for you to search for health-related information on the Internet.

12. **When looking for health information on the internet, how easy or difficult is it for you...**

12.1 **... to choose the right words or queries to find the information you are looking for?**

- ○ Very easy
- ○ Easy
- ○ Difficult
- ○ Very difficult
- ○ I don't know

12.2 **... to find precisely the information you are looking for?**

- ○ Very easy
- ○ Easy
- ○ Difficult
- ○ Very difficult
- ○ I don't know

12.3 **... to understand the information found?**

- ○ Very easy
- ○ Easy
- ○ Difficult
- ○ Very difficult
- ○ I don't know

12.4 **... to judge the reliability of the information found?**

- ○ Very easy
- ○ Easy
- ○ Difficult
- ○ Very difficult
- ○ I don't know

12.5 **... to determine whether commercial interests are hidden behind the information offered?**

- ○ Very easy
- ○ Easy
- ○ Difficult
- ○ Very difficult
- ○ I don't know

12.6 **... to consult several websites to compare the information they offer on the same subject?**

- ○ Very easy
- ○ Easy

○ Difficult
○ Very difficult
○ I don't know

12.7 **... to determine if the information found is relevant to you?**

○ Very easy
○ Easy
○ Difficult
○ Very difficult
○ I don't know

12.8 **... to use the information found to try to solve a health problem?**

○ Very easy
○ Easy
○ Difficult
○ Very difficult
○ I don't know

13. **When you type a health-related message on a digital device, how easy or difficult is it for you...**

13.1 **... to clearly formulate a written message that you send to a health provider?** (e.g. your question, description of the situation or personal information)

○ Very easy
○ Easy
○ Difficult
○ Very difficult
○ I don't know

13.2 **… to express your opinion, your thoughts, your feelings or to ask a question in writing on social networks (including online forums)?**

○ Very easy
○ Easy
○ Difficult
○ Very difficult
○ I don't know

We are going to ask you a few questions about the Electronic Patient Record (EPR) itself.

> **→ If you have <u>already opened</u> an Electronic Patient Record, continue with the following questions.**
>
> **→ If you <u>have not opened</u> an Electronic Patient Record, go directly to question 23.**

14. **What are your motivations for opening a EPR?**

Several answers are possible.

- ☐ Have access to my health documents (e.g. monitoring of my health)
- ☐ Better know my health and my treatments (e.g. medications)
- ☐ Improve the quality and safety of my care, especially in the event of an emergency
- ☐ Make better choices about my health
- ☐ Share my health documents with my family or friends
- ☐ Share my health documents with my doctor/other healthcare professional
- ☐ Other

15. **Have you opened the EPR yourself?**

- ○ Yes
- ○ a representative I chose from my family opened it for me

16. **How often have you consulted your EPR over the last three months?**

- ○ Never
- ○ 1 time
- ○ 2 times or more regularly

17. **Have you submitted any documents in your EPR?**

- ○ Yes, by myself
- ○ Yes, a representative did it for me
- ○ No

18. **Are you satisfied with the use of your EPR today?**

- ○ Yes
- ○ No
- ○ I don't know

If not, why? ____

19. **Do you think that the EPR has an effect on your state of anxiety?**

- O Yes
- O No
- O I don't know

If so, your state of anxiety, since the opening of the EPR...

- O has improved a lot
- O has improved
- O has remained unchanged
- O has deteriorated
- O has deteriorated considerably

20. **Do you think that the EPR improves the quality and safety of your health care?**

- O Yes
- O No
- O I don't know

21. **Do you think that the EPR has an effect on the quality of the relationship with your doctor or other health professional?**

- O Yes
- O No
- O I don't know

If so, the quality of the relationship with your doctor or other health professional...

- O has improved a lot
- O has improved
- O has remained unchanged
- O has deteriorated
- O has deteriorated considerably

22. **Would you recommend an EPR to a loved one?**

- O Yes
- O No
- O I don't know

If not, why? ____

Thank you for your answers. Please continue to question 26.

23. **Are you aware of the Electronic Patient Record (EPR) offered in Switzerland?**

○ Yes
○ No
○ I don't know

24. **What are the reasons why you have not yet opened an EPR?**

Several possible answers.

☐ I lack information
☐ I find the process too complicated, or I didn't do it alone
☐ I don't have time
☐ I don't find any personal interest in it
☐ I fear a personal data leak
☐ Other

If "other": For what other reason have you not yet opened an EPR?

____

25. **What would be your motivations for opening an EPR?**

Several possible answers.

☐ Have access to my health documents (e.g. for monitoring or control)
☐ Better know my health and treatments (e.g. medications)
☐ Improve the quality and safety of my care, especially in the event of an emergency
☐ Make better choices about my health
☐ Share my health documents with my family or friends
☐ Share my health documents with my doctor/other health care professional
☐ I don't see any.

We are going to ask you a few questions related to your medical network.

26. **Do you have a general practitioner?**

○ Yes
○ No

27. **How many health professionals have you consulted in the last 3 months?**

○ None
○ One
○ Two or more

28. **Have you visited an emergency department in the last 3 months?**

○ Yes
○ No

We end with some general questions about your health.

29. **How do you assess your overall health?**

- ○ Very good
- ○ Good
- ○ Medium
- ○ Poor
- ○ Very poor

30. **Are you currently affected by a chronic or long-term illness (you are taking regular medication and/or have been monitored for this disease for 6 months or more).**

- ○ Yes
- ○ No
- ○ I don't know

Interview and follow-up study on the same topic

31. **Do you agree that the research team can contact you again to offer you a 30-minute interview?**

    ○ Yes
    ○ No

    If yes: please leave your contact details on the following page to be contacted according to your preferences (p. 17).

32. **Do you agree that the research team may contact you again to participate in a new study on the same theme?**

    ○ Yes
    ○ No
