# Supplementary material for: Usage and Perceptions of Electronic Patient Records Experienced by Users and Nonusers in the Canton of Vaud, Switzerland: Mixed Methods Study
Source: J Med Internet Res. 2026 Apr 21;28:e83702. doi: 10.2196/83702 (PMC13107525; doi:10.2196/83702)
Supplement: Multimedia Appendix 2 [file jmir-v28-e83702-s002.pdf]

APPENDIX B: INTERVIEW GUIDE FOR PATIENTS

Socio-demographic data: age, self-identified gender, profession, education level

Level	Content	Question
Technology	Utilization of digital tools in day-to-day activities	• How do you use technology tools in your daily life?<br>• Do you consider yourself to be a 'connected' person?<br>○ If yes, for what reasons? If not, why not?<br>○ And what about your loved ones?<br>• Which tools do you use most often?
Opening of an electronic patient record (EPR)	Motivation<br><br>Context<br><br>Process	• What were the main reasons for opening it?<br>○ Incentives?<br>○ Connection to your health status?<br>• Could you tell me how you opened the EPR?<br>• How was the process of opening the EPR?<br>○ Did you receive any support?<br>○ What worked well?<br>○ What improvements could be made?<br>• What are your expectations for the development of the EPR?<br>○ In terms of EPR content?<br>○ In terms of medical history?<br>• What are your recommendations regarding opening the EPR?<br>○ How can more people be reached to use the EPR?
Utilisation of EPR	Consultations<br><br>Informations regarding your health status<br><br>Coordination between health and social care professionals<br><br>Impact on informal caregivers	• In your opinion, what is the general use of the EPR for the population?<br>• Could you tell me how you have used it or how you intend to use it?<br>○ Regarding information about your health status?<br>○ Regarding the use of this information?<br>○ For which professionals ?<br>• How would you rate the coordination between providers?<br>○ With or without EPR use ?<br>○ Do you think coordination between providers will be affected? If so, how? If not, why not?<br>• Could the use of the EPR have an impact on your family?<br>○ If so, how?<br>○ If not, please explain
Experiences of EPR during consultations	Information exchange<br><br>Interaction	• What is your experience with the EPR?<br>• What types of information are recorded?<br>• What type of interaction do you have with providers?<br><br>**Communication**

	Communication<br><br>Collaboration	• Do you think that the use of the EPR influences communication with your healthcare professionals? If so, how?<br>• What do you think about consultations/interactions by phone, video, etc.?<br>○ Do you think there will be information missing from the EPR?<br><br>**Use of digital tools for healthcare**<br><br>• Do you use other tools (YouTube videos, apps, etc.) to supplement your care?<br>• Are you confident in using them?<br>• Do you know how to sort through relevant information from websites?<br><br>In general, what would be your EPR user recommendations during consultations?
